# Statistical Methods in the Analysis of the Effect of Carbonisate on the Hardness of Epoxy-Resin-Based Composites

**DOI:** 10.3390/ma17235916

**Published:** 2024-12-03

**Authors:** Agata Wieczorska, Sebastian Drewing

**Affiliations:** Faculty of Marine Engineering, Gdynia Maritime University, Morska St. 81-87, 81-225 Gdynia, Poland; s.drewing@wm.umg.edu.pl

**Keywords:** recycling, pyrolysis, carbonisate, statistical analysis, hardness

## Abstract

This research concerns the manufacture and characterisation of epoxy composites with the addition of carbonisate, obtained by the pyrolysis of MDF (medium-density fibreboard) furniture board waste. The laminated composites were made by hand lamination, with the carbonisate used as a filler to improve the mechanical properties of the composite. The carbonisate was obtained by the thermal decomposition of MDF waste in an anaerobic environment by pyrolysis, which is an efficient method of waste management and material recycling. The resulting carbonisate was integrated into an epoxy resin matrix to investigate its potential as a reinforcing agent. The article describes a study on the hardness of epoxy-resin-based composites to which carbonisate was added in different fractions and percentages. The aim of the research was to test the possibility of using char as a component in improving the mechanical properties of epoxy composites with a view towards creating a durable recycled material with optimal parameters. As part of the study, a statistical analysis of the results of hardness measurements was carried out to accurately assess the effect of the quantity and size of the carbonate particles on the mechanical properties of the materials. The analysis identified significant differences between samples and verified the repeatability of the results. It was found that the addition of carbonisate to the A0 base sample (without the addition of carbonisate) leads to a significant hardening of the material. This was confirmed by the higher medians of samples A01 (carbonisate 5% with a 0.5 mm fraction), A02 (carbonisate 7.5% with a 0.5 mm fraction), and A03 (carbonisate 5% with a 1.0 mm fraction) compared to the base sample. The most homogeneous hardness was shown in sample A02, with the highest concentration of results and the lowest values of standard deviation and spread. The results indicated that the addition of carbonate significantly increased the hardness of the composite materials, with optimal stability achieved at 7.5% (by weight) of carbonate with a 0.5 mm fraction. The conducted research precisely determined the influence of the amount and characteristics of carbonisate particles on the mechanical properties of the materials, which enables the more effective designing of future composites. The statistical results provide a reliable basis for evaluating the potential applications of these materials in various industrial sectors, such as construction, automotive and aerospace, where high hardness and durability are important.

## 1. Introduction

Epoxy-resin-based composites are of growing interest in a wide range of industries due to their exceptional mechanical and thermal and chemical resistance properties. A variety of additives, such as glass fibres, carbon fibres, nanoparticles, and various mineral and organic fillers, are used to further improve these materials and tailor their properties to specific applications. These additives can significantly affect the strength, hardness, wear resistance, thermal and electrical conductivity of the composites. Choosing the right additives and their proportions allows the properties of composites to be modified, making them more versatile and effective in various applications such as automotives, aerospace, construction, and electronics [1,2,3]. Technological processes that allow the production of epoxy composites, such as resin infusion and lamination, enable the manufacture of components with a wide variety of shapes and sizes, further increasing their attractiveness in industry [4,5,6,7]. This article focuses on evaluating the effect of additives, such as carbonisate, on the mechanical properties, particularly hardness, of epoxy composites.

The authors’ study [8] analysed the hardness and impact strength of an epoxy resin matrix reinforced with banana fibre and biocomposites containing *Camellia sinensis* particles. The experimental results indicated that the addition of *Camellia sinensis* particles increases the hardness of the epoxy composites with banana fibre while decreasing the impact energy and impact strength of these materials. In contrast, the authors of [9] conducted a study on the effect of sisal fibre treatment on the impact strength and hardness of epoxy-resin-based composites. It was found that the hardness of composites with sisal fibres, both treated and untreated, increases gradually with an increasing fibre content, reaching a maximum value at a weight proportion of 10%. An article [10] analysed the effect of graphite content on the mechanical properties of polyester–glass recyclate composites used as structural components. The materials were made by hand lamination with 10% recyclate and nano-added graphite. The specimens were subjected to a static tensile test according to the standard and the results showed that increasing the graphite content reduced the properties of the composite, albeit disproportionately to the amount of graphite. Another paper [11] investigated how silica nanoparticles (SiO_2_) affect the mechanical properties of an epoxy resin. The addition of nano-silica significantly improves the hardness, tensile strength, and impact resistance of the resin. Another research [12] focused on the wear resistance and surface morphology of multi-walled carbon-nanotube filled (MWCNT) epoxy composites. In this study, the effects of different concentrations of MWCNTs (0 wt%, 0.25 wt%, 0.50 wt% and 0.75 wt%) on the wear properties of these composites were analysed. This research not only added to the knowledge of the wear mechanisms of bio-based epoxy composites but was also part of the growing emphasis on sustainable materials. Another study [13] analysed how graphene nanoplatelets affect the hardness and strength of epoxy composites. The addition of graphene improved both hardness and wear resistance, making the composites more resistant to mechanical loads. Article [14] investigates the effect of fly ash as a low-cost filler on the hardness and mechanical properties of epoxy composites. Fly ash increases hardness and impact resistance at relatively low production costs. Another paper [15] focuses on the synergy between glass fibre and silica nanoparticles in epoxy composites. The results show that both forms of reinforcement significantly improve the hardness and strength of the composites. The authors of [16] investigated the mechanical properties, tribological properties (related to friction, wear, and lubrication), and surface morphology of glass-fibre reinforced composites that were enriched with hybrid fillers of ilmenite and silicon dioxide (SiO_2_). A study [17] investigated the effect of zinc oxide (ZnO) nanoparticles on the mechanical properties and hardness of epoxy composites. ZnO improves both hardness and resistance to external agents such as UV. An article [18] evaluated the effect of mineral and natural additives (2.5; 5; 10 wt%) on the impact strength of epoxy–basalt composites. Three types of fillers were used to modify the epoxy matrix: basalt powder (BP), basalt microfibre (BF), and sunflower husk ash (SA). The results of the study confirmed that the addition of powder fillers to the epoxy matrix of basalt-fibre-reinforced composites is an effective method to improve their impact characteristics. Liu, W. and others [19] presented a strategy for the design of multi-walled oligomeric silsesquioxane toughening agents and their application in epoxy resin, demonstrating an effective method for toughening the resin matrix of advanced composites. Abramczyk, N. et al. [20] have shown that the addition of 2% gamma aluminium nanopowder slightly reduces the hardness of a pure polyester–glass composite but the same addition allows the hardness of composite materials to be increased by the addition of glass recyclate.

The research on composites with the addition of carbonisate obtained by pyrolysing MDF boards is part of a sustainable approach to the design of building materials, which is reflected in the work of Mahmoud Sodangi et al. [21] and Elisabetta Negro et al. [22]. According to the approach presented by Sodangi, the use of carbonisate can be an example of an innovative material that helps overcome barriers to implementing sustainable solutions in the construction industry by providing alternatives to conventional materials based on non-renewable raw materials. Similarly, research by Elisabetta Negro et al. [22] highlights that the use of sustainable and innovative building materials is crucial not only for energy efficiency but also for improving the structural integrity of buildings. Composites with carbonisate can find applications in building renovation, especially where solutions with a low environmental impact and high durability are required. The pyrolysis process of MDF enables the efficient processing of waste, which further supports the idea of a closed-loop economy, important in the context of sustainable construction. Thus, research on composites with carbonisate can add to the existing literature, providing both innovative tools for analysis and practical solutions to foster the implementation of sustainable materials in construction.

The research carried out by many authors highlights the importance of various additives and modifications in optimising the mechanical properties of composites, which is crucial for their applications in the construction and materials industry. Continued research in this area can contribute to a better understanding of the interactions between the different components of composites and to the development of modern, more sustainable materials. Analyses on composites show that the introduction of different fillers, nanoparticles, and other modifiers significantly affects key mechanical properties such as hardness, impact strength, and wear resistance.

An analysis of the literature has shown that carbonisate has not been used so far as a filler for epoxy composites. The rationale for the study was based on the need to precisely determine how the number and characteristics of carbonisate particles affect the mechanical properties of the material, allowing for the better design of future composites. The use of carbonisate in epoxy composites has a definite positive aspect, especially if the pyrolysis process is carried out using renewable energy. This type of approach promotes waste recycling, reduces pressure on natural resources, and contributes to reducing emissions.

## 2. Materials and Methods

The material used in the study comprised glass–epoxy laminates with carboniser. Carbon black obtained from the pyrolysis of MDF is characterised by a high carbon content of 79.17%, including ash 6.95%, nitrogen 4.43%, hydrogen 2.99%, chlorine 0.08%, and sulphur 0.07%. EM 1002/450/125 emulsion matting with random fibre direction was used as reinforcement for the carbonisate composites. The matrix was epoxy resin Epidian 6, and hardener Z-1 was used; this was applied to the resin used. The simplest method of forming composite elements is by hand lamination. It involves placing reinforcement in a mould (successive layers of glass mat) and saturating its layers with resin using rollers, brushes, etc. The next step was to determine the proportion of resin, mat, and carbonisate in order to saturate the entire mat and obtain a material with the best properties. The following test material was prepared: glass mat A0 (10 layers) base sample without carboniser, A01 (10 layers) with the addition of 5% carboniser of 0.5 mm fraction, A02 (10 layers) with the addition of 7.5% carboniser of 0.5 mm fraction, and sample A03 (10 layers) with the addition of 5% carbonier of 1.0 mm fraction. Table 1 summarises the content of each composite material made by hand lamination.

**Figure 1:**
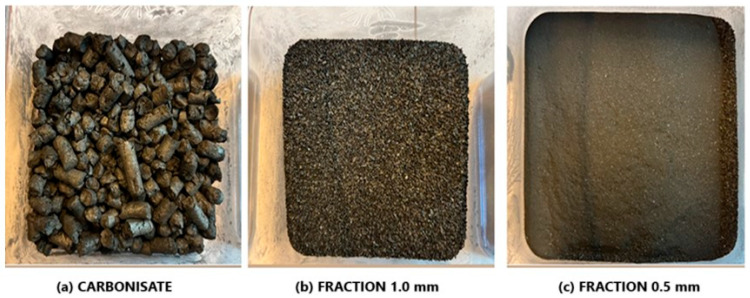
Carbonisate (**a**) after pyrolysis, (**b**) after crushing and screening to a 1.0 mm fraction, and (**c**) after crushing and screening to a 0.5 mm fraction.

Carbonisate obtained from the pyrolysis of furniture waste, including MDF, is distinguished by its large fraction. It has a well-developed porosity, but in order to improve the mechanical properties of the composite, including hardness, it must have the right morphology (e.g., small particles with a regular shape). This fraction was carefully crushed and sieved using a sieve shaker to obtain specific particle sizes, as shown in Figure 1.

Hardness measurements of plastics are carried out for both design and research purposes, as well as for product quality assurance. Hardness tests were carried out using the Barcol method [23]. In this method, measurement is carried out using a stylus which, under spring load, is pressed into a sample placed on a stable support, and a reading is taken on a scale of 1 to 100. According to the standards for composite materials, 30 measurements were taken for each sample.

**Figure 2:**
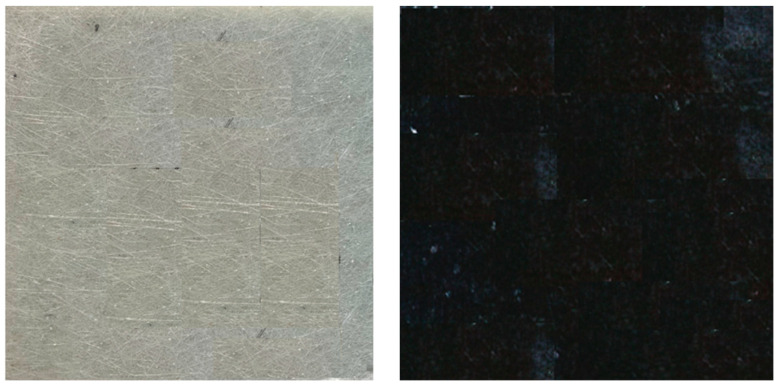
Preparation samples for hardness tests without and with carboniser added.

Specimens of 200 × 200 mm were prepared, as shown in Figure 2. A total of 90 hardness measurements were taken for the sample without carboniser A0; the higher number of measurements for the sample without carboniser was due to the need for obtaining accurate reference data while 30 hardness measurements were taken for the other samples A01, A02, and A03.

**Figure 3:**
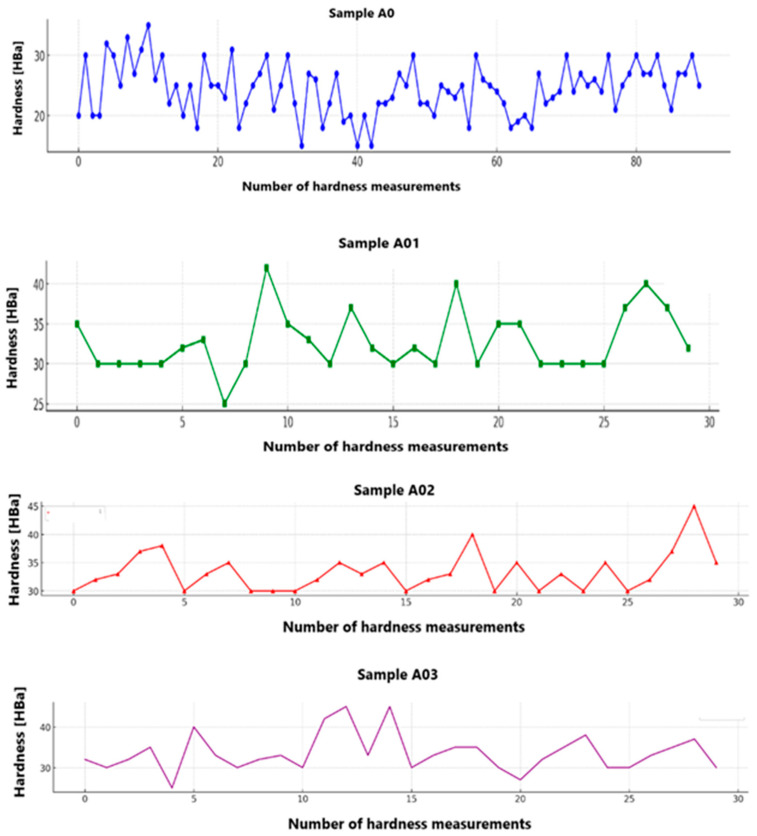
Hardness measurements for samples A0 (blue graph), A01 (green graph), A02 (red graph), and A03 (purple graph).

Table 2 shows the average hardness values of the tested composite materials with the use of the Barcol hardness tester. Figure 3 summarises the hardness measurements for the specimens made.

Average measurement hardness tests showed that sample A02 had the highest level of hardening compared both to the base sample A0 and to the other samples A01 and A03.

As part of the study, a statistical analysis of the results of the hardness measurements was carried out with the aim of gaining a more accurate understanding of the effect of the amount and size of the carbonate fraction on the mechanical properties of the materials.

Determination of sample size:

The Barcol 934-1 hardness tester manual for fibreglass-reinforced plastics indicates that the number of measurements for hardness 30 on the HBa scale is 29, according to GB/T 3854-2005.

According to the instructions, a baseline sample (*n* = 30) was conducted for sample A0. An analysis was then undertaken to determine whether the sample size specified in the instructions corresponded to that calculated based on Stein’s computational model, assuming that the general population had a normal distribution or otherwise [24].

(1) $$n\geq \frac{t_{\alpha ,n-1\;}^{2}S_{\left(x\right)}^{2}}{d^{2}}$$

Here, *t* is read value of the *t*–student distribution, *α* is the significance level, *n* − 1 is number of degrees of freedom, *S* is the standard deviation, and *d* is the maximum allowable measurement error.

To determine the sample size, for A0 material, parametric Student’s *t*-tests and ANOVA were used for statistical analyses. We calculated the following: *S* = 2.763472744 (for the first 30 baseline measurements taken of sample A0), *d* = 1, and *t* = 2.0452 for the significance level *α* = 95%. According to Formula (1), the sample size *n* was 31.9434588 and was similar to that indicated in the instruction manual of the device. The value from the instruction *n* = 30 was used for further studies.

## 3. Results

Statistical methods were used in the analysis of the strength parameters of epoxy–glass composites modified with carbonisate.

### 3.1. Testing Normality of Distributions: Shapiro–Wilk Test and Chi-Square Test for Small Sample Sizes

The chi-square test [25] of concordance is the most commonly used non-parametric test. It is used to verify the hypothesis that an observed characteristic X in the general population has a specific type of distribution, e.g., binomial, Poisson, normal, etc. The Shapiro–Wilk test [26] is considered the best test for checking the normality of the distribution of a random variable. The main strength of this test is its high power, i.e., for a fixed α, the probability of rejecting the hypothesis H0 if it is false is higher than for other such tests. The calculated statistical values are summarised in Table 3.

Assumed α = 95%.

The null and alternative hypotheses are of the following forms:

**H0.**  *The distribution of the trait under study is a normal distribution;*

**H1.**  *The distribution of the trait under study is not a normal distribution.*

Conclusion: The obtained results of both the chi-square as well as the Shapiro–Wilk test probability values showed the following:

- For samples A0, A01, and A02, the obtained results of the probability values of *p* were smaller than 0.05; therefore, the null hypothesis had to be rejected and the alternative hypothesis had to be accepted, i.e., the hardness variables of the tested samples were not in normal distributions.
- For specimen A03, the obtained probability values *p* were greater than 0.05; therefore, there were no grounds to reject the null hypothesis. The hardness variables for all test samples were in normal distributions.

Conclusion: Parametric tests cannot be used to test differences, so non-parametric tests will be used in further analyses [27].

### 3.2. ANOVA Kruskal–Wallis Test and Median Test

It was decided to check whether the measurements taken for the basic/benchmark sample A0 were similar to each other, i.e., whether they came from the same population. A total of 90 hardness measurements were taken for sample A0 and then randomly combined into three pairs (N = 3, with samples labelled A0, A01, and A02) with a sample size of *n* = 30. The three samples were then compared to each other to see if there were any significant statistical differences between them. The study variables in the samples did not have normal distributions, so the non-parametric statistics ‘ANOVA Kruskal-Wallis test and median test’ were used to perform the tests. The Kruskal–Wallis one-way analysis of variance for ranks, or Kruskal–Wallis ANOVA, is an extension of the Mann–Whitney U-test to more than two independent populations. This test is used to verify the hypothesis of non-significance of differences between the medians of the variable under study in several population groups (whereby we assume that the distributions of the variable are close to each other). The null hypothesis is that the measures of the position (distribution) of the trait under study are the same in all the groups being compared:

- **H0:** *F1 = F2 = F3; all samples come from the same unit;*
- **H1:** *F1 ≠ F2 ≠ F3; not all samples come from the same units.*

**Figure 4:**
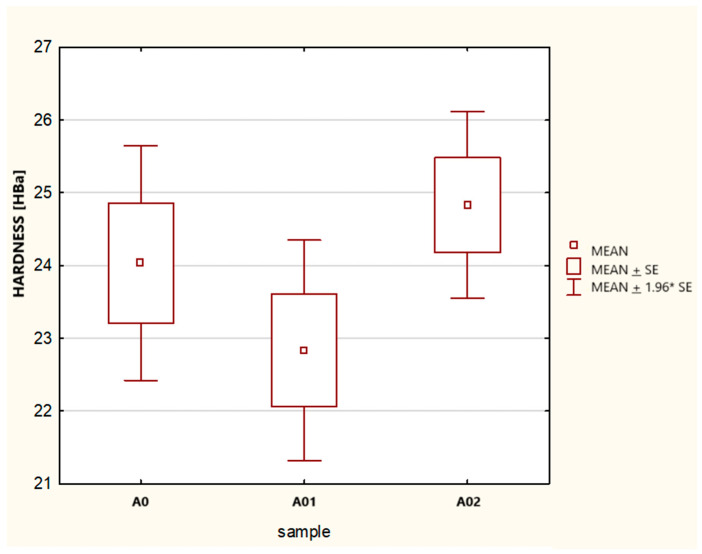
Box-and-whisker plot and chi-square median test for samples A0, A01, A02,where mean ± SE indicates mean ± standard error and mean ± 1.96 × SE is confidence interval for the mean.

The obtained results (Figure 4, Table 4) of both the Kruskal–Wallis ANOVA test as well as the chi-square median test showed that there were no significant statistical differences between the samples, with the calculated *p* = 0.2048 being greater than the assumed minimum significance threshold of pv = 0.05. This demonstrated the repeatability of the measurement series performed, with all three samples (measurement series) coming from a single population, and this was the basis for making further comparisons in the configurations of standard/basic sample A0 and samples with carbonate admixtures A01, A02, and A03.

### 3.3. Testing for Differences Non-Parametric Statistics Kruskal–Wallis ANOVA of Test Samples

Due to the lack of ‘normality’, or non-normality of the distributions and the inequality of variance, of the hardness values, it was decided to perform non-parametric tests for them: the ‘ANOVA Kruskal -Wallis test and median test’. Samples A0, A01, A02, and A03 were used for the comparisons, with sample A0 randomly limited to 30.

The null hypothesis that the tested measures of the trait position (distribution) in all compared groups were the same was accepted:

- **H0:** *F1 = F2 = F3 = F4, all samples come from the same population;*
- **H1:** *F1 ≠ F2 ≠ F3≠4, not all samples are from the same population.*

**Figure 5:**
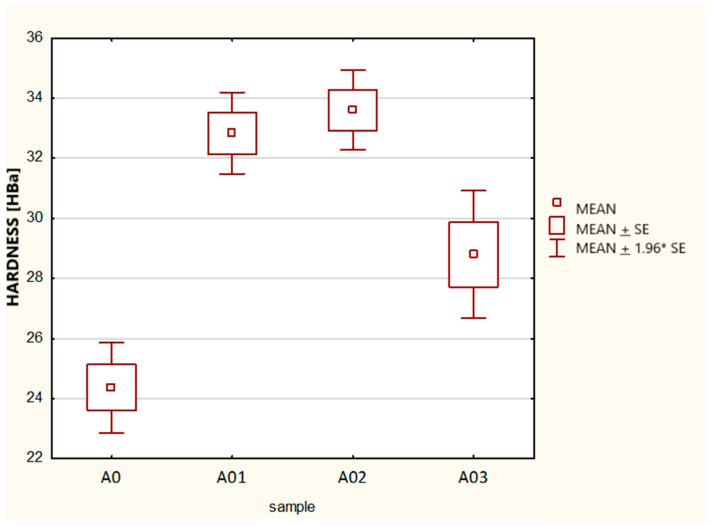
Box-and-whisker diagram for the materials tested for samples A0, A01, A02 and A03, where mean ± SE indicates mean ± standard error and mean ± 1.96 × SE is confidence interval for the mean.

The results obtained (Table 5) of both the Kruskal–Wallis ANOVA test as well as the chi-square median test (Figure 5) showed the following:

- There were no significant statistical differences between test samples A01 and A02. For the test samples, the obtained probability *p*-values were greater than 0.05, and therefore, there were no grounds to reject the null hypothesis.
- For the tested samples in the configurations, sample A0 and samples A01, A02, and A03, the obtained results of the probability values *p* were lower than 0.05, and therefore, there were no grounds to accept the null hypothesis. Samples A01, A02, and A03 were different from sample A0.

## 4. Interpretation of the Results Obtained

**Figure 6:**
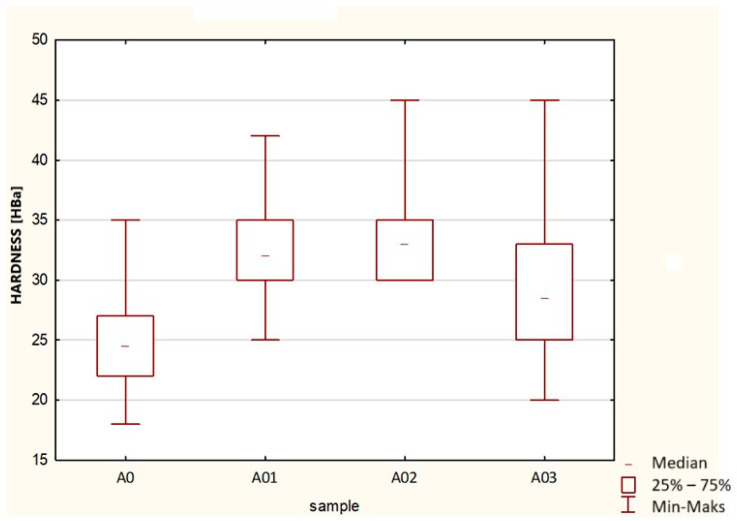
Box-and-whisker diagrams for the hardness of the materials tested.

Based on the analysis of the results presented in Figure 6 (frame-and-whisker diagram) and Table 6, the following conclusion can be drawn.

The addition of carbonisate to sample A0 leads to an increase in the hardness of the material, which has been confirmed by the higher median values in samples A01, A02, and A03. Of these, sample A02 shows the most stable and uniform hardness properties, characterised by the smallest range of results, the smallest standard deviation, and the highest values of the first and third quartiles. Although sample A03 achieves a higher median hardness than sample A0, its range of results significantly overlaps with the values of sample A0, which may indicate a lower hardness repeatability in this sample. Sample A02 seems to be the most optimal in terms of stability and the predictability of mechanical properties.

Conclusion: The material corresponding to sample A02 shows the greatest hardening and stability of this parameter.

## 5. Conclusions

The results of the hardness measurements showed that the addition of carbonisate significantly improved the hardness of the composites. Each sample containing carbonisate achieved a higher hardness compared to the base sample. Preliminary hardness measurements indicated that the greatest increase in hardness was observed in sample A02, which reached a value of 33.6 HBa. Statistical analysis confirmed that the addition of carbonisate significantly affected the hardening of the material, as reflected by higher median hardness values. In addition, the use of the Kruskal–Wallis test allowed the comparison of the ranges and deviations, indicating sample A02 as the most homogeneous in terms of hardness.

The research conducted indicates the great potential of carbonisate as an additive to increase the hardness of epoxy composites, especially in the context of industrial waste recycling. In the future, it is worth focusing on the further optimisation of the composition and structure of these materials to achieve the best combination of mechanical properties such as hardness, abrasion resistance, and resistance to external agents. The development of carbonisate composites for new industries such as automotives, aerospace, and construction, where materials with high strength and durability are required, is also an important direction. Sustainability and a closed-loop economy are key areas for further research, including the search for efficient methods of recycling composites containing carbonisate. In addition, the integration of carbonisate with other materials, such as nanomaterials, may enable new functional properties of composites such as electrical or thermal conductivity.

## Figures and Tables

**Table 1:** Content of individual composite materials made by hand lamination.

No.	Fraction Carbonisate [mm]	Number of Layers Mats	Content Resin % (by Weight)	Content Mats % (by Weight)	Content Carbonisate % (by Weight)	Determination Samples
1	-	10	60	40	0	A0
2	0.5	10	60	35	5	A01
3	0.5	10	60	32.5	7.5	A02
4	1.0	10	60	35	5	A03

**Table 2:** Hardness of composite materials (average measurement).

Label of the Sample	Hardness HBa
A0	23.9
A01	32.8
A02	33.6
A03	28.8

**Table 3:** Calculated values of the Shapiro–Wilk test statistic and the chi-square test for the samples tested.

Label of the Sample	Shapiro–Wilka Value *p*	Chi-Square Test Value *p*
A0	0.04986	0.03796
A01	0.00663	0.01832
A02	0.00041	0.03942
A03	0.17321	0.33887

**Table 4:** Kruskal–Wallis ANOVA test *p*-values.

Label of the Sample	*p*-Value for Multiple Comparisons; Hardness (Barcol Hardness Measurement), Independent (Grouping) Kruskal–Wallis Test: H (2, N = 90) = 3.171471 *p* = 0.2048)		
	A0	A01	A02
A0	-	1.00000	0.891275
A01	1.00000	-	0.235614
A02	0.891275	0.235614	-

**Table 5:** Kruskal–Wallis ANOVA test *p*-values—non-parametric statistics.

Label of the Sample	*p*-Value for Multiple Comparisons; Hardness (Barcol Hardness Measurement), Independent (Grouping) Kruskal–Wallis Test: H (3, N = 120) = 50.90029 *p* = 0.0000			
	A0	A01	A02	A03
A0	-	0.000000	0.000000	0.029944
A01	0.000000	-	1.000000	0.027138
A02	0.000000	1.000000		0.002853
A03	0.029944	0.027138	0.002853	-

**Table 6:** Summary values of test samples.

Parameter	Label of the Sample			
	A0 (*n* = 90)	A01 (*n* = 30)	A02 (*n* = 30)	A03 (*n* = 30)
standard deviation—σ	4.19	3.80	3.71	5.92
Variance—σ^2^	17.55	14.42	13.77	35.06
Median	24.50	32.00	33.00	28.50
Mean	24.37	32.83	33.60	28.80
Minimum	18.00	25.00	30.00	20.00
Maximum	35.00	42.00	45.00	45.00
Range	17.00	17.00	15.00	25.00
Third quartile (Q3 or Q75)	26.75	35	35	33
First quartile (Q1 or Q25)	22	30	30.25	25

## Data Availability

The data presented in this study are available on request from the corresponding author.
